# Pooling-analysis on hMLH1 polymorphisms and cancer risk: evidence based on 31,484 cancer cases and 45,494 cancer-free controls

**DOI:** 10.18632/oncotarget.21810

**Published:** 2017-10-10

**Authors:** Sha Li, Yi Zheng, Tian Tian, Meng Wang, Xinghan Liu, Kang Liu, Yajing Zhai, Cong Dai, Yujiao Deng, Shanli Li, Zhijun Dai, Jun Lu

**Affiliations:** ^1^ Clinical Research Center, First Affiliated Hospital of Xi’an Jiaotong University, Xi’an, Shaanxi 710061, China; ^2^ Department of Pharmacy, Second Affiliated Hospital of Xi’an Jiaotong University, Xi’an, Shaanxi 710004, China; ^3^ Department of Oncology, Second Affiliated Hospital of Xi’an Jiaotong University, Xi’an, Shaanxi 710004, China

**Keywords:** hMLH1, polymorphism, cancer, meta-analysis

## Abstract

To elucidate the veritable relationship between three hMLH1 polymorphisms (rs1800734, rs1799977, rs63750447) and cancer risk, we performed this meta-analysis based on overall published data up to May 2017, from PubMed, Web of knowledge, VIP, WanFang and CNKI database, and the references of the original studies or review articles. 57 publications including 31,484 cancer cases and 45,494 cancer-free controls were obtained. The quality assessment of six articles obtained a summarized score less than 6 in terms of the Newcastle-Ottawa Scale (NOS). All statistical analyses were calculated with the software STATA (Version 14.0; Stata Corp, College Station, TX). We found all the three polymorphisms can enhance overall cancer risk, especially in Asians, under different genetic comparisons. In the subgroup analysis by cancer type, we found a moderate association between rs1800734 and the risk of gastric cancer (allele model: OR = 1.14, P = 0.017; homozygote model: OR = 1.33, P = 0.019; dominant model: OR = 1.27, P = 0.024) and lung cancer in recessive model (OR = 1.27, P = 0.024). The G allele of rs1799977 polymorphism was proved to connect with susceptibility of colorectal cancer (allele model: OR = 1.21, P = 0.023; dominate model: OR = 1.32, P <0.0001) and prostate cancer (dominate model: OR = 1.36, P <0.0001). Rs63750447 showed an increased risk of colorectal cancer, endometrial cancer and gastric cancer under all genetic models. These findings provide evidence that hMLH1 polymorphisms may associate with cancer risk, especially in Asians.

## INTRODUCTION

As one of the pivotal pathways in maintaining genetic stability, MMR system is mainly in charge of repairing the replication-associated errors, including removing mistaken bases, correcting substitutions and rectifying insertion-deletion mismatches. Its defects may result in microsatellite instability (MSI), a type of genetic instability related to colorectal cancer, gastric cancer, and endometrial cancer, etc. [1–3] Interest in MLH1 has increased in the last few years because MLH1 was discovered as a key component in MMR for MSI, and its dysfunction is supposed to be implicated in cancer predisposition.

MLH1 not only takes part in the activities of MMR system, but also has other interesting cellular functions, such as participating in cell cycle arrest, triggering DNA damage-induced apoptosis to response to some chemical or physical agents [4], and interacting with tumor-related signaling molecules like BRCA1 [5] and p53 [6]. Moreover, various polymorphisms were found in MLH1 gene, part of them were proved to influence the expression of functional MLH1. We selected three most common loci rs1800734, rs1799977, and rs63750447 in hMLH1 which may alter the function of the hMLH1 gene according to literature. Among these, the A allele of rs1800734 polymorphism could alter the methylation level of nine CpG sites mapped on the MLH1 promoter [7], while rs1799977 and rs63750447 were situated at the exons of hMLH1 [1, 8]. Emerging inspiring evidences indicate these functional polymorphisms of hMLH1 may be potential candidates in mediating hereditary susceptibility to cancer, however, applying them in clinical application is still treated critically. Past decades witnessed numerous molecular epidemiological studies carried out worldwide to investigate the actual association between them, yet no coincident conclusion was reached so far.

For example, Nizam et al. [9] concluded that rs1800734 polymorphism had an influence on colorectal cancer (CRC) risk among Malaysians in 2013, while Zhang *et al* [10] found no obvious connection between rs1800734 and CRC risk in 2016. For rs1799977 polymorphism, Milanizadeh et al. [11] detected it could increase CRC risk particularly in female patients, but Peng *et al.* [1] hold a contrary opinion that no association existed between the two. The inconsistent conclusions also existed in the studies exploring the relationship between hMLH1 polymorphisms and other cancer types. Although rs63750447 polymorphism was accepted as a risk factor for east-Asian CRC patients [1, 12–14], no reliable conclusion reported on the possible relationship between rs63750447 and overall cancer or other kinds of tumors. To solved these controversies, a comprehensive and persuasive meta-analysis was excepted to conduct depending on complete published data and proper methodological tools, thus we carried out this meta-analysis to illuminate the objective connection between hMLH1 polymorphisms (rs1800734, rs1799977 and rs63750447) and cancer risk.

## RESULTS

### Characteristics of eligible studies

**Figure 1:**
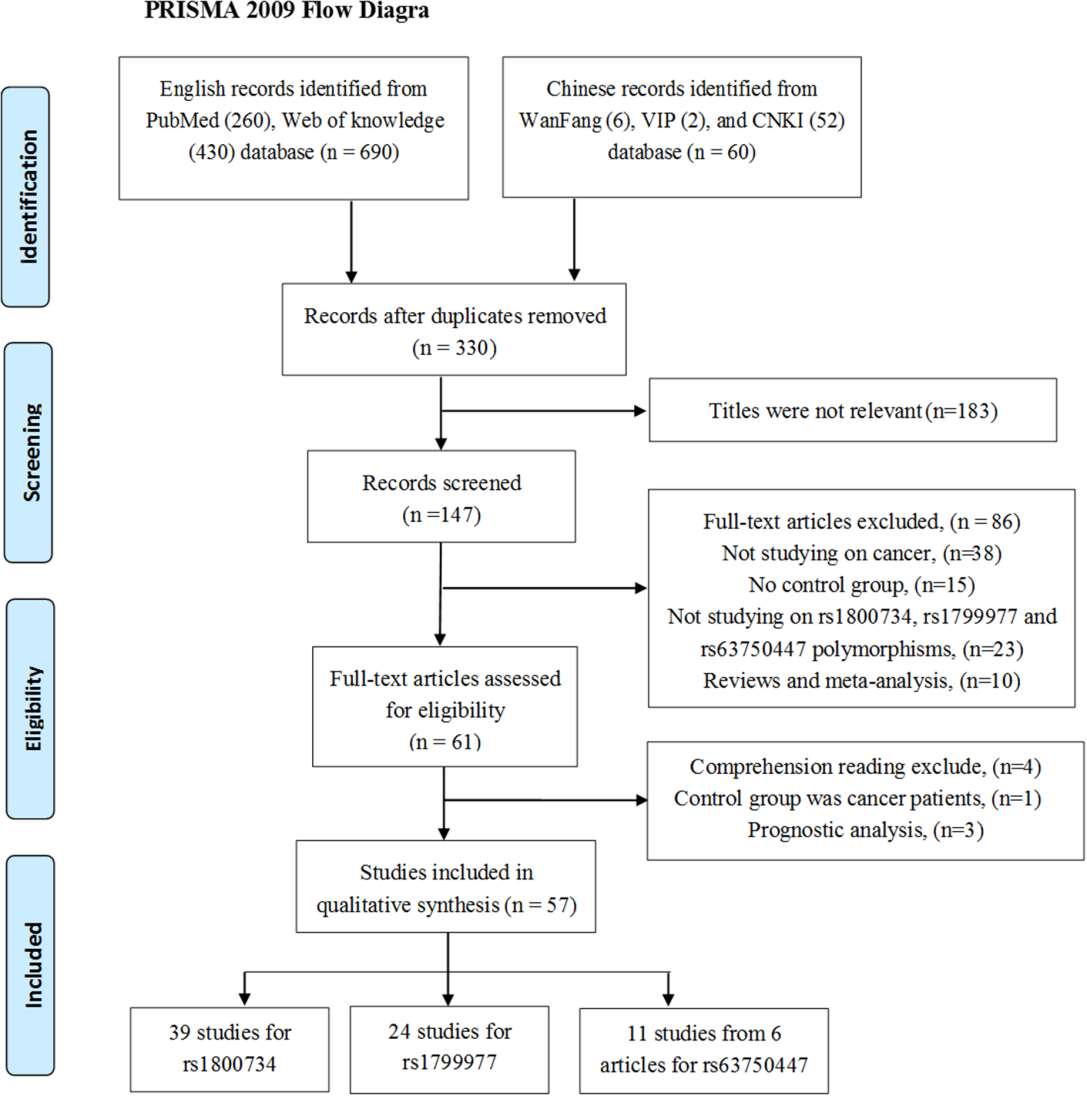
The flow diagram of the meta-analysis, according to the PRISMA 2009 CNKI = China National Knowledge Infrastructure.

Finally, we obtained a total of 57 publications including 31,484 cancer cases and 45,494 cancer-free controls (all were from the databases and no study was identified by manual search of the references of the original studies or review articles). The detail selection process was shown in the flow diagram (Figure 1). What needed illustration is that we abandon three studies contained in previous meta-analyses after comprehensive reading full text. The first one was the study performed by Chen *et al* [15], contained in the meta-analyses conducted in 2011 [16] and 2015 [17], which was excluded on account of both its cases group and controls group are women with cancers (cases with MLH1 methylation while controls not). Another study finished by van Roon *et al*. [18], also included in previous meta-analyses [17, 19], has two controls groups collected from literature [20, 21]. We excluded it after discussing with a senior author within us. And the third study we abandoned was due to deficiency of cancer-free control group [16].

Among the 57 eligible literatures, 26 were based on Caucasian background from, Poland, Spain, the United States, Denmark, the United Kingdom, Sweden, Portugal, Czech Republic and Canada. 27 were carried out in Asians from China, Kazakhstan, India, Iran, Malaysia, Japan and Korea, and four were based on mixed ethnic groups. All the publications involving rs63750447 polymorphism were carried out among the Chinese population. Three case-cohort designed studies [22–24] and 54 case-controlled studies were involved in this meta-analysis. All cancer cases were confirmed by pathology or histology, involved cancer types covering colorectal, gastric, ovarian, head and neck, endometrium, lung, bladder, prostate, thyroid, breast, prostate, Non-Hodgkin lymphoma, acute myeloid leukaemia, and acute lymphoblastic leukaemia. The quality assessment of six studies obtained a summarized score less than 6 in terms of the Newcastle-Ottawa Scale (NOS), four of them are studying on rs1800734 [25–28] while one of them is for rs63750447 [29], and the other one focused on rs1800734 and rs1799977 polymorphisms [30]. Specially, two publications by Zhang et al. [8] and Wang et al. [29] contained four and three independent studies respectively. One study focused on rs1799977 polymorphism by Joshi et al. [31] did not provide complete genotype frequencies. Hence only the dominant model was evaluated. Detail characteristics of eligible publications are displayed in Table 1.

**Table 1:** Characteristics of the studies included in the meta-analysis

First author	Year	Country	Ethnic	Method	Control	Disease	SNP	NOS
Peng [1]	2016	China	Asian	PCR-HRM	Population	CRC	2, 3	7
Zhang [10]	2016	China	Asian	TaqMan	Hospital	CRC	1	6
Zhu [2]	2016	China	Asian	TaqMan	Population	GC	1	7
Djansugurova [46]	2015	Kazakhstan	Asian	PCR-RFLP	Hospital	CRC	1	8
Niu [47]	2015	China	Asian	PCR-RFLP	Hospital	OC	1, 2	6
Nogueira [48]	2015	Brazil	Mixed	TaqMan	Hospital	HNSCC	1	6
Poplawski [3]	2015	Poland	Caucasian	PCR-RFLP	Hospital	EC	1	6
Slovakova [49]	2015	Slovak	Caucasian	PCR-RFLP	Population	LC	1	8
Rodriguez [50]	2014	Spain	Caucasian	PCR-RFLP	Hospital	BT	1	6
Jha [51]	2013	India	Asian	PCR-RFLP	Population	HNSCC	1	7
Martinez-Uruena [25]	2013	Spain	Caucasian	PCR-RFLP	Hosptal	CRC	1	4
Milanizadeh [11]	2013	Iran	Asian	PCR-RFLP	Hospital	CRC	2	7
Nizam [9]	2013	Malaysia	Asian	PCR-RFLP	Hospital	CRC	1	6
Muniz-Mendoza [30]	2012	Mexico	Mixed	PCR-RFLP	Hospital	CRC	1, 2	4
Savio [32]	2012	Canada	Caucasian	PCR-RFLP	Population	CRC	1	7
Xiao [52]	2012	China	Asian	PCR	Population	GC	1, 2	8
Zhi [53]	2012	China	Asian	PCR-RFLP	Population	BLC	1	7
Lacey [54]	2011	Poland	Caucasian	iSelect bead chip	Population	EC	1, 2	8
Lo [55]	2011	China	Asian	PCR	Hospital	LC	1	7
Soni [56]	2011	India	Asian	TaqMan	Hospital	PC	1	6
Whiffin [57]	2011	UK	Asian	KASPae	Population	CRC	1	8
Zhi [58]	2011	China	Asian	PCR-RHM	Hospital	GC	1	6
Langeberg [59]	2010	USA	Caucasian	ABI	Population	PC	2	7
Picelli [22]	2010	Sweden	Caucasian	Direct sequencing	Population	CRC	2	7
Shi [12]	2010	China	Asian	PCR	Hospital	TC	1, 2, 3	6
Campbell [41]	2009	USA	Caucasian	PCR-RFLP	Population	CRC	1, 2	8
Conde [37]	2009	Portugal	Caucasian	QIAamp	Hospital	BC	2	6
Joshi [31]	2009	USA	Caucasian	TaqMan	Population	CRC	2	7
Nejda [38]	2009	Spain	Caucasian	PCR-RFLP	Hospital	CRC	2	7
Ohsawa [13]	2009	Japan	Asian	PCR-RFLP	Unknown	CRC	3	6
Shih [33]	2009	China	Asian	PCR-RFLP	Population	LC	1	7
Tanaka [60]	2009	Japan	Asian	Direct sequencing	Population	PC	2	7
An [61]	2008	China	Asian	PCR-RFLP	Population	LC	1, 2	8
Christensen [23]	2008	Denmark	Caucasian	SBE-tags	Population	CRC	2	8
Harlay [26]	2008	Canada	Mixed	MassARRAY	Hospital	OC	1	5
Koessler [62]	2008	UK	Caucasian	TaqMan	Population	CRC	1	7
Samowitz [20]	2008	USA	Caucasian	Direct sequencing	Population	CRC	1	7
Scott [34]	2008	UK	Caucasian	TaqMan	Population	NHL	1	6
Tulupova [63]	2008	Czech	Caucasian	TaqMan	Hospital	CRC	1	7
Worrillow [64]	2008	UK	Caucasian	PCR-RFLP	Population	AML	1	6
Berndt [24]	2007	USA	Caucasian	TaqMan	Population	CRC	2	8
Raptis [21]	2007	Canada	Caucasian	TaqMan	Population	CRC	1, 2	7
Beiner [35]	2006	Canada	Mixed	MassARRAY	Hospital	EC	1	6
Landi [65]	2006	Mixed	Caucasian	PCR	Hospital	LC	2	7
Mei [14]	2006	China	Asian	PCR	Hospital	CRC	2, 3	6
Song [39]	2006	Mixed	Caucasian	TaqMan	Population	OC	1, 2	6
Chen [66]	2005	China	Asian	PCR-RFLP	Hospital	HCC	1	7
Lee [67]	2005	Korea	Caucasian	MassARRAY	Hospital	BC	1	6
Kim [68]	2004	Korea	Asian	TaqMan	Population	CRC	2	6
Listgarten [40]	2004	Canada	Caucasian	QIAmp	Hospital	BC	2	6
Park [36]	2004	Korea	Caucasian	PCR	Population	LC	1	8
Zhang [8]	2004	China	Asian	DHPLC	Population	Mixed	3	7
Deng [69]	2003	China	Asian	DHPLC	Hospital	GC	1	7
Mathonnet [70]	2003	Canada	Caucasian	PCR-ASO	Population	ALL	2	6
Shin [27]	2002	Korea	Asian	PCR-SSCP	Hospital	CRC	1	4
Wang [29]	2000	China	Asian	PCR-SSCP	Hospital	Mixed	3	5
Ito [28]	1999	Japan	Asian	PCR-SSCP	Hospital	CRC	1	4

### Quantitative synthesis

The distributions of genotypes frequencies of hMLH1 polymorphisms (rs1800734; rs1799977; rs63750447) for every single study are exhibited in Table 2. The minor allele frequencies (MAF) among cancer cases varied widely according to the included studies, ranging from 0.205 to 0.656 for rs1800734 polymorphism, 0.016 to 0.744 for rs1799977 polymorphism, and 0.032 to 0.069 for rs63750447 polymorphism. The average MAF of case-group for the three polymorphisms is 0.396, 0.233, 0.053, respectively. The meta-analysis results of these three polymorphisms were shown in Supplementary Table 1.

**Table 2:** Genotype distribution and allele frequency of hMLH1 polymorphisms

First author	Genotype (N)								Allele frequency (N)				MAF	HWE
	Case (n)				Control (n)				Case (n)		Control (n)			
	total	AA	AB	BB	total	AA	AB	BB	A	B	A	B		
**-93G>A (rs1800734)**														
Zhang 2016 [10]	312	66	139	107	300	52	154	94	271	353	258	342	0.566	0.414
Zhu 2016 [2]	406	49	213	144	444	79	235	130	311	501	393	495	0.617	0.125
Niu 2015 [47]	421	51	188	182	689	150	356	183	290	552	656	722	0.656	0.348
Djansugurova 2015 [46]	249	126	94	29	244	101	115	28	346	152	317	171	0.305	0.581
Nogueira 2015 [48]	450	248	171	31	450	269	159	22	667	233	697	203	0.259	0.809
Poplawski 2015 [3]	100	18	81	1	100	9	50	41	117	83	68	132	0.415	0.254
Slovakova 2015 [49]	422	250	144	28	511	260	228	23	644	200	748	274	0.237	0.002
Rodriguez 2014 [50]	115	61	44	10	200	115	79	6	166	64	309	91	0.278	0.080
Jha 2013 [51]	245	52	90	100	205	98	79	28	194	290	275	135	0.599	0.067
Martinez-Uruena2013 [25]	383	233	131	19	236	129	102	5	597	169	360	112	0.221	0.003
Nizam 2013 [9]	104	22	50	32	104	33	33	38	94	114	99	109	0.548	0.000
Muniz-Mendoza2012 [30]	100	47	44	9	115	39	55	21	138	62	133	97	0.310	0.835
Savio 2012 [32]	252	150	96	6	845	528	264	53	396	108	1320	370	0.214	0.012
Xiao 2012 [52]	554	104	262	188	588	124	271	193	470	638	519	657	0.576	0.113
Zhi 2012 [53]	311	43	163	105	302	41	161	100	249	373	243	361	0.600	0.059
Larcy 2011 [54]	414	251	141	22	404	241	146	17	643	185	628	180	0.223	0.381
Lo 2011 [55]	719	235	344	140	728	256	366	106	814	624	878	578	0.434	0.177
Soni 2011 [56]	105	44	40	21	106	27	61	18	128	82	115	97	0.390	0.101
Whiffin 2011 [57]	10409	6408	3504	497	6965	4395	2261	309	16320	4498	11051	2879	0.216	0.401
Zhi 2011 [58]	236	36	111	89	240	42	114	84	183	289	198	282	0.612	0.757
Shi 2010 [12]	204	40	102	62	204	34	99	71	182	226	167	241	0.554	0.959
Campbell 2009 [33]	1600	952	553	95	1963	1170	688	105	2457	743	3028	898	0.232	0.769
Shih 2009 [33]	165	41	64	60	193	36	113	44	146	184	185	201	0.558	0.016
An 2008 [61]	500	163	243	94	517	169	258	90	569	431	596	438	0.431	0.618
Harley 2008 [26]	842	483	297	62	776	532	206	38	1263	421	1270	282	0.250	0.003
Koessler 2008 [62]	2288	1407	778	103	2276	1392	777	107	3592	984	3561	991	0.215	0.914
Samowitz 2008 [20]	1006	610	344	52	1963	1170	688	105	1564	448	3028	898	0.223	0.769
Scott 2008 [34]	601	375	205	21	942	610	310	22	955	247	1530	354	0.205	0.016
Tulupova 2008 [63]	619	359	216	44	611	365	209	37	934	304	939	283	0.246	0.336
Worrillow 2008 [64]	390	246	128	16	918	585	292	41	620	160	1462	374	0.205	0.554
Raptis 2007 [21]	929	554	331	44	1098	687	352	59	1439	419	1726	470	0.226	0.118
Beiner 2006 [35]	654	377	220	57	764	524	202	38	974	334	1250	278	0.255	0.002
Song 2006 [39]	1306	825	414	67	1951	1224	638	89	2064	548	3086	816	0.210	0.615
Chen 2005 [66]	545	86	261	198	374	85	178	111	433	657	348	400	0.603	0.400
Lee 2005 [67]	783	201	348	234	594	117	292	185	750	816	526	662	0.521	0.927
Park 2004 [36]	372	66	176	130	371	71	206	94	308	436	348	394	0.586	0.027
Deng 2003 [69]	54	8	27	19	56	9	29	18	43	65	47	65	0.602	0.636
Shin 2002 [27]	139	33	61	45	157	42	74	41	127	151	158	156	0.543	0.473
Ito 1999 [28]	27	8	10	9	84	22	46	16	26	28	90	78	0.519	0.355
**655A>G(rs1799977)**														
Peng2016 [1]	156	151	5	0	311	307	4	0	307	5	618	4	0.016	0.909
Niu 2015 [47]	418	383	33	2	689	613	75	1	799	37	1301	77	0.044	0.406
Milanizadeh 2013 [11]	219	25	62	132	248	54	119	75	112	326	227	269	0.744	0.599
Muniz-Mendoza 2012 [30]	102	71	26	5	100	81	19	0	168	36	181	19	0.176	0.294
Xiao 2012 [52]	554	522	31	1	592	568	23	1	1075	33	1159	25	0.030	0.143
Larcy 2011 [54]	417	210	160	47	406	196	165	45	580	254	557	255	0.305	0.253
Langeberg 2010 [59]	1251	578	555	118	1236	607	514	115	1711	791	1728	744	0.316	0.681
Picelli 2010 [22]	1781	819	781	181	1701	832	708	161	2419	1143	2372	1030	0.321	0.560
Shi 2010 [12]	204	185	17	2	204	192	11	1	387	21	395	13	0.051	0.072
Campbell 2009 [41]	1601	764	678	159	1944	937	848	159	2206	996	2722	1166	0.311	0.087
Conden 2009 [37]	287	129	129	29	546	255	251	40	387	187	761	331	0.326	0.039
Joshi 2009 [31]	301	161	/	/	354	194	/	/	/	/	/	/	/	/
Nejda 2009 [38]	140	41	72	27	125	64	44	17	154	126	172	78	0.450	0.044
Tanaka 2009 [60]	177	159	16	2	131	120	11	0	334	20	251	11	0.056	0.616
An 2008 [61]	500	479	20	1	504	493	11	0	978	22	997	11	0.022	0.804
Christensen 2008 [23]	380	172	170	38	770	364	327	79	514	246	1055	485	0.324	0.661
Berndt 2007 [24]	211	100	94	17	2090	968	896	226	294	128	2832	1348	0.303	0.387
Raptis 2007 [21]	929	451	391	87	1098	514	485	99	1293	565	1513	683	0.304	0.310
Landi 2006 [65]	291	145	123	23	309	129	151	29	413	169	409	209	0.290	0.107
Mei 2006 [14]	160	144	14	2	150	141	9	0	302	18	291	9	0.056	0.705
Song 2006 [39]	1022	507	418	97	1224	624	477	123	1432	612	1725	723	0.299	0.026
Kim 2004 [68]	107	100	7	0	330	311	18	1	207	7	640	20	0.033	0.192
Listgarten 2004 [40]	170	89	64	17	156	76	75	5	242	98	227	85	0.288	0.008
Mathonnet 2003 [70]	287	149	112	26	320	154	132	34	410	164	440	200	0.286	0.474
**1151T>A(rs63750447)**														
Peng2016 [1]	156	142	13	1	311	310	1	0	297	15	621	1	0.048	0.977
Shi 2010 [12]	204	178	24	2	204	191	12	1	380	28	394	14	0.069	0.108
Ohsawa 2009 [13]	670	630	39	1	332	327	5	0	1299	41	659	5	0.031	0.890
Mei 2006 [14]	160	142	18	0	150	141	9	0	302	18	291	9	0.056	0.705
Zhang 2004 (EC) [8]	233	206	27	0	268	251	17	0	439	27	519	17	0.058	0.592
Zhang 2005 (CRC) [8]	90	82	8	0	268	251	17	0	172	8	519	17	0.044	0.592
Zhang 2004 (BC) [8]	111	104	7	0	268	251	17	0	215	7	519	17	0.032	0.592
Zhang 2004 (GC) [8]	273	240	33	0	268	251	17	0	513	33	519	17	0.060	0.592
Wang 2000 (CRC) [29]	101	88	13	0	100	94	6	0	189	13	194	6	0.064	0.757
Wang 2000 (EC) [29]	76	69	7	0	100	94	6	0	145	7	194	6	0.046	0.757
Wang 2000 (GC) [29]	79	68	11	0	100	94	6	0	147	11	194	6	0.070	0.757

A: the major allele, B: the minor allele, MAF: minor allele frequencies; HWE: Hardy–Weinberg equilibrium.

### Rs1800734 polymorphism

**Figure 2:**
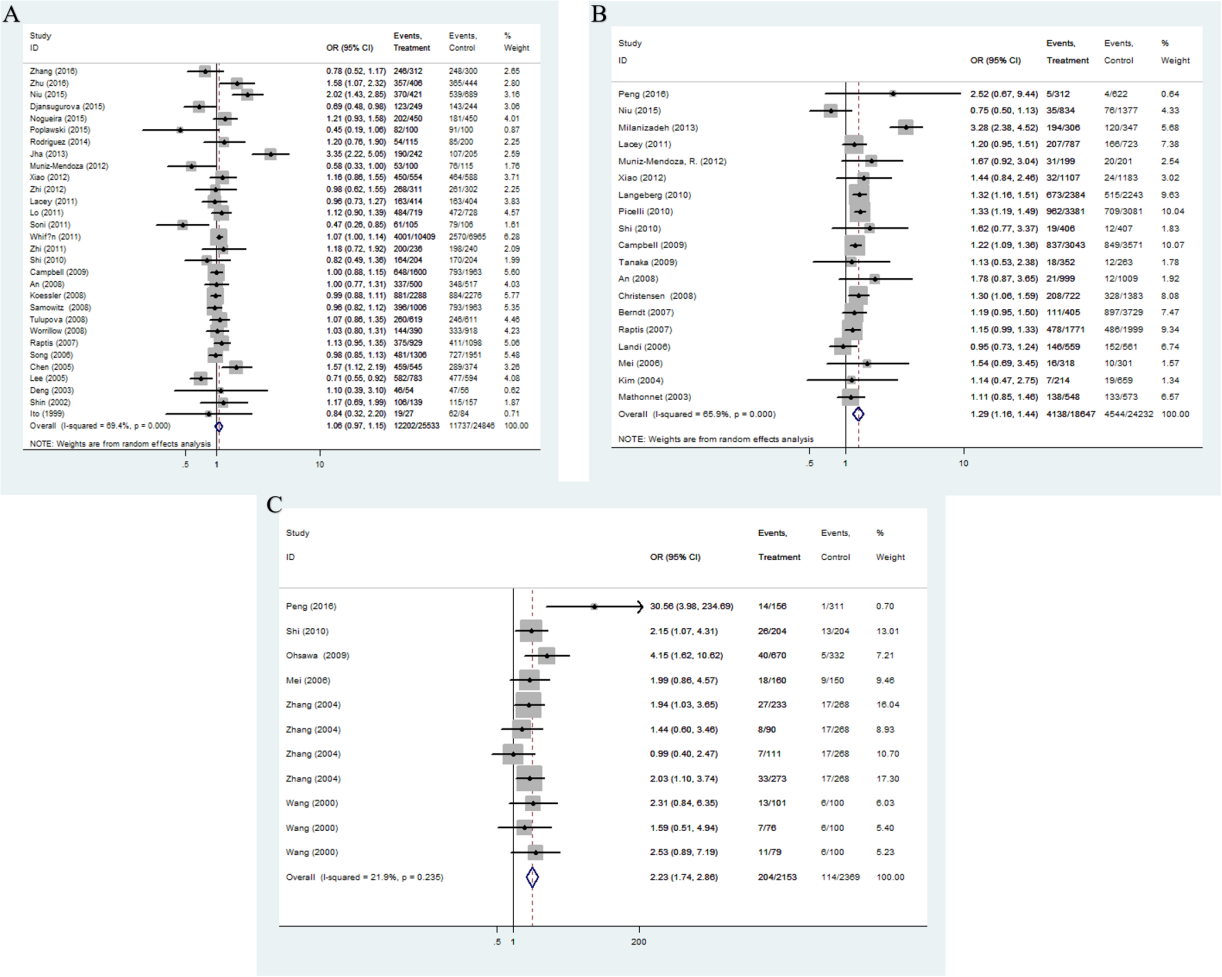
Forest plot of OR with 95%CI for the hMLH1 polymorphisms with cancer risk under dominate model according to HWE (**(A)** rs1800734; **(B)** rs1799977; **(C)** rs63750447). CI: confidence interval, OR: odds ratio, HWE: Hardy-Weinberg equilibrium.

Overall, there are 39 studies including 29,331 cases and 29,588 controls for rs1800734 polymorphism. Statistically significance was found between rs1800734 polymorphism and overall cancer risk under five genetic models (recessive comparison: OR = 1.22, 95%CI = 1.09-1.37, *P* = 0.001; homozygote comparison: OR = 1.23, 95%CI = 1.06-1.42, *P* = 0.006; allele comparison: OR = 1.08, 95%CI = 1.01-1.16, *P* = 0.023). After excluding nine studies that were not in accordance with HWE [3, 9, 25, 26, 32–36], we observed increased risks of all kinds of cancers under two genetic models (recessive comparison: OR = 1.18, 95%CI = 1.04-1.34, *P* = 0.012; homozygote comparison: OR = 1.18, 95%CI = 1.00-1.39, P = 0.048, Figure 2A).

**Figure 3:**
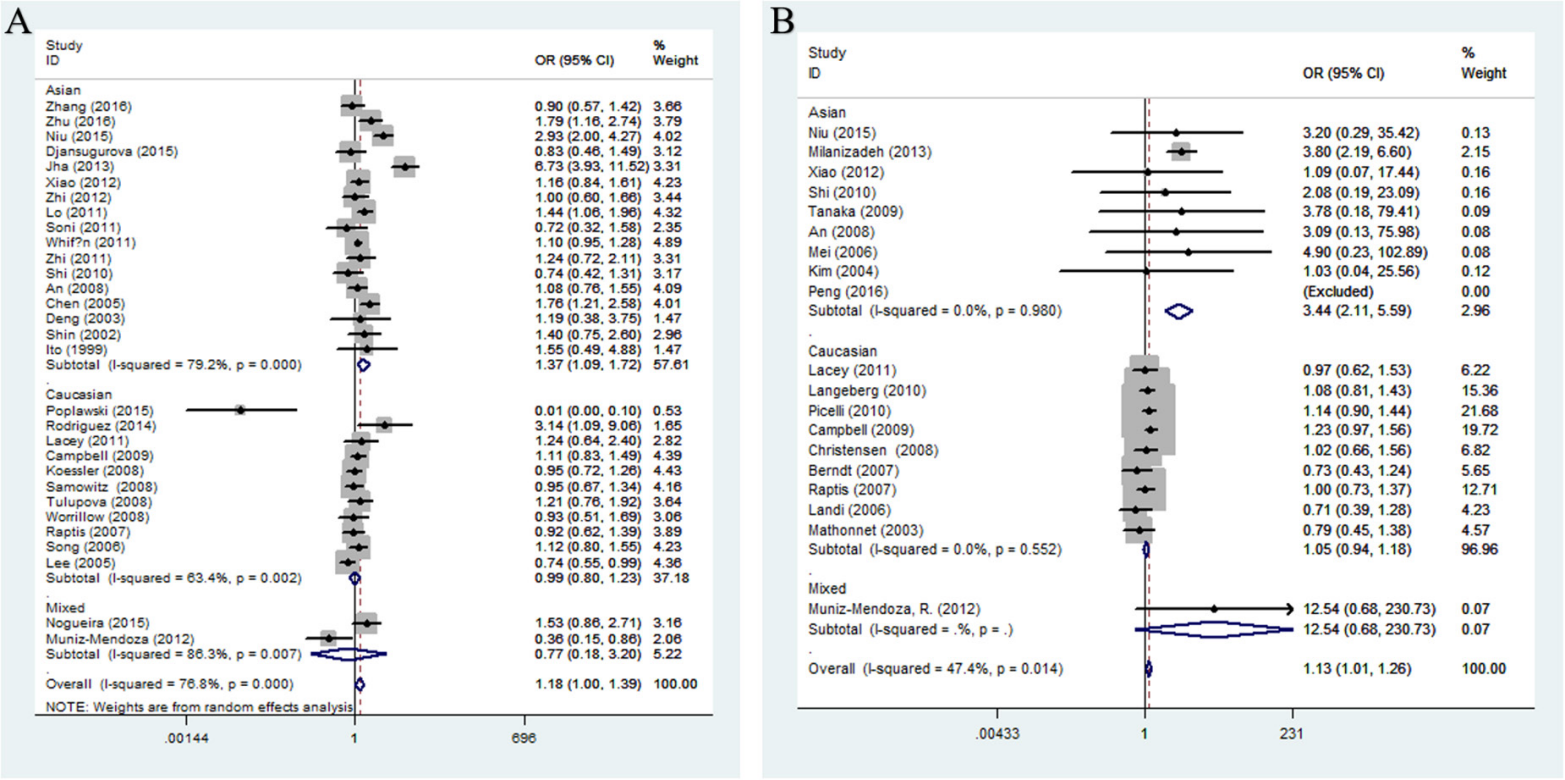
Stratified analysis by ethnicity for the association between hMLH1 polymorphisms and cancer risk under homozygote model according to HWE (**(A)** rs1800734; **(B)** rs1799977). CI: confidence interval, OR: odds ratio, HWE: Hardy-Weinberg equilibrium.

In the stratification analysis based on ethnicity (Figure 3A), we found no association between cancer risk and Caucasian population, while the mutation allele A contributed to an increasing cancer risk in Asian population under three comparison models (recessive comparison: OR = 1.30, 95%CI = 1.11-1.53, *P* = 0.001; homozygote comparison: OR = 1.37, 95%CI = 1.09-1.72, *P* = 0.006; allele comparison: OR = 1.16, 95%CI = 1.03-1.31, *P* = 0.014). In the cancer-specific analysis, rs1800734 polymorphism showed a potential tendency to enhance gastric and lung cancer susceptibility in different genetic comparisons (gastric cancer: dominate comparison: OR = 1.27, 95%CI = 1.03-1.56, *P* = 0.024; homozygote comparison: OR = 1.33, 95%CI = 1.06-1.68, *P =* 0.019, allele comparison: OR = 1.14, 95%CI = 1.02-1.28, *P* = 0.017; lung cancer: recessive comparison: OR = 1.27, 95%CI = 1.03-1.57, *P* = 0.024). Besides, the subgroup analysis depended on the source of controls suggested us that rs1800734 polymorphism had an influence on cancer risk under four genetic models among population-based controls (dominate comparison: OR = 1.05, 95%CI = 1.01-1.10, *P* = 0.016, recessive comparison: OR = 1.12, 95%CI = 1.04-1.22, *P* = 0.004; homozygote comparison: OR = 1.22, 95%CI = 1.00-1.49, *P =* 0.050; heterozygous comparison: OR = 1.05, 95%CI = 1.01-1.10, *P* = 0.031; allele comparison: OR = 1.10, 95% = 1.00-1.20, *P* = 0.041) and recessive comparison among hospital-based controls (OR = 1.27, 95%CI = 1.03-1.57, *P* = 0.024). And, when the subgroup analysis was conducted based on a quality score, rs1800734 polymorphism displayed an increased cancer risk among high-quality studies, but no association was found among low-quality studies (Supplementary Table 1).

### Rs1799977 polymorphism

We finally derived 11,665 cases and 15,538 controls from 24 eligible studies for rs1799977 polymorphism. All the studies obtained high-quality scores according to the Newcastle-Ottawa Scale (NOS). In general, we found the variant G allele of rs1799977 could improve overall cancer risks under three genetic models (dominant comparison: OR = 1.28, 95%CI = 1.16-1.41, *P* < 0.0001; homozygote comparison: OR = 1.15, 95%CI = 1.04-1.27, *P* = 0.006; allele comparison: OR = 1.12, 95%CI = 1.02-1.23, *P* = 0.017). After excluding four studies [37–40] that were not in accordance with HWE (Figure 2B), the pooled ORs and 95%CI revealed a possible increased risk of cancer (dominant comparison: OR = 1.25, 95%CI = 1.18-1.33, *P* < 0.0001; homozygote comparison: OR = 1.13, 95%CI = 1.01-1.26, *P* = 0.027).

**Figure 4:**
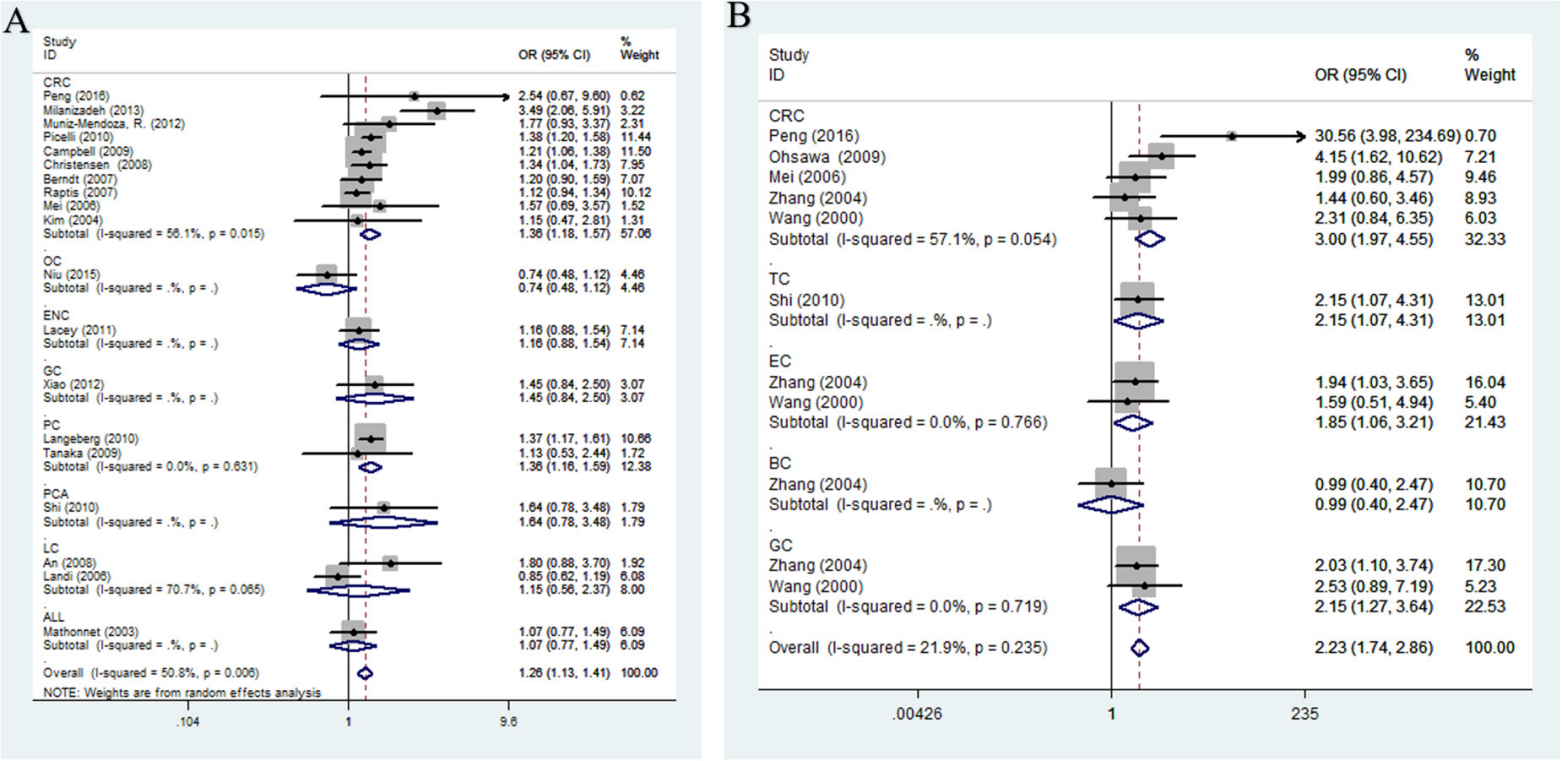
Stratified analysis by cancer type for the association between hMLH1 polymorphisms and cancer risk under dominant model according to HWE (**(A)** rs1799977; **(B)** rs63750447). CI: confidence interval, OR: odds ratio. CRC: colorectal cancer; GC: gastric cancer; BC: breast cancer; PC: prostate cancer; EC: endometrial cancer; OC: ovarian carcinoma; GC: gastric cancer; LC: lung cancer; other: other cancer; HWE: Hardy-Weinberg equilibrium.

When the subgroup carried out by ethnicity (Figure 3B), a significant association was observed between rs1799977 and cancer risk among Asians in four genetic models (dominant comparison: OR = 1.52, 95%CI = 1.04-2.24, *P* = 0.033; recessive comparison: OR = 3.34, 95%CI = 2.33-4.78, *P* < 0.0001; homozygote comparison: OR = 3.44, 95%CI = 2.12-5.59, *P* < 0.0001; allele comparison: OR = 1.64, 95%CI = 1.38-1.95, *P* < 0.0001) and Caucasians in only dominant model (OR = 1.24, 95%CI = 1.16-1.32, *P* < 0.0001). In the cancer-specific analysis (Figure 4A), rs1799977 polymorphism showed a correlation between colorectal cancer under two genetic models (dominant comparison: OR = 1.32, 95%CI = 1.16-1.51, *P* < 0.0001; allele comparison: OR = 1.21, 95%CI = 1.03-1.42, *P* = 0.023) and prostate cancer under dominant model (OR = 1.36, 95%CI = 1.16-1.59, *P* < 0.0001).

Besides, the results of subgroup analyses by source of control and study design exhibited in the Supplementary Table 1.

### Rs63750447 polymorphism

A total of 2153 cancer cases and 1365 cancer-free controls from 11 studies were involved in our meta-analysis for rs63750447 polymorphism. Since the homozygous mutant AA of rs63750447 polymorphism was in very rare frequencies, we chose allele model, heterozygous model and dominant model to evaluate the association strength. The pooled analysis observed a significant association between cancer risk and rs63750447 polymorphism (dominant comparison: OR = 2.23, 95%CI = 1.75-2.86, *P* < 0.0001; heterozygote comparison: OR = 2.21, 95%CI = 1.73-2.84, *P* < 0.0001; allele comparison: OR = 2.19, 95%CI = 1.72-2.78, *P* < 0.0001), as shown in Figure 2C.

The subgroup analysis by cancer type (Figure 4B) indicated that rs63750447 polymorphism had influences on colorectal cancer (dominant comparison: OR = 2.87, 95%CI = 1.42-5.82, *P* = 0.003; heterozygote comparison: OR = 2.81, 95%CI = 1.42-5.57, *P* = 0.003; allele comparison: OR = 2.84, 95%CI = 1.38-5.81, *P* = 0.004), gastric cancer (dominant comparison: OR = 2.15, 95%CI = 1.27-3.64, *P* = 0.005; heterozygote comparison: OR = 2.2115, 95%CI = 1.27-3.64, *P* = 0.005; allele comparison: OR = 2.19, 95%CI = 1.24-3.47, *P* = 0.006), and endometrium cancer (dominant comparison: OR = 2.23, 95%CI = 1.06-3.21, *P*= 0.029; heterozygote comparison: OR = 1.85, 95%CI = 1.06-3.21, *P* = 0.029; allele comparison: OR = 1.80, 95%CI = 1.05-3.09, *P* = 0.033). When we conducted the subgroup analysis by quality score, there was a significantly increased cancer risk for rs63750447 polymorphism in both high-quality studies and low-quality studies (shown in Supplementary Table 1).

### Test of heterogeneity and sensitivity analysis

**Figure 5:**
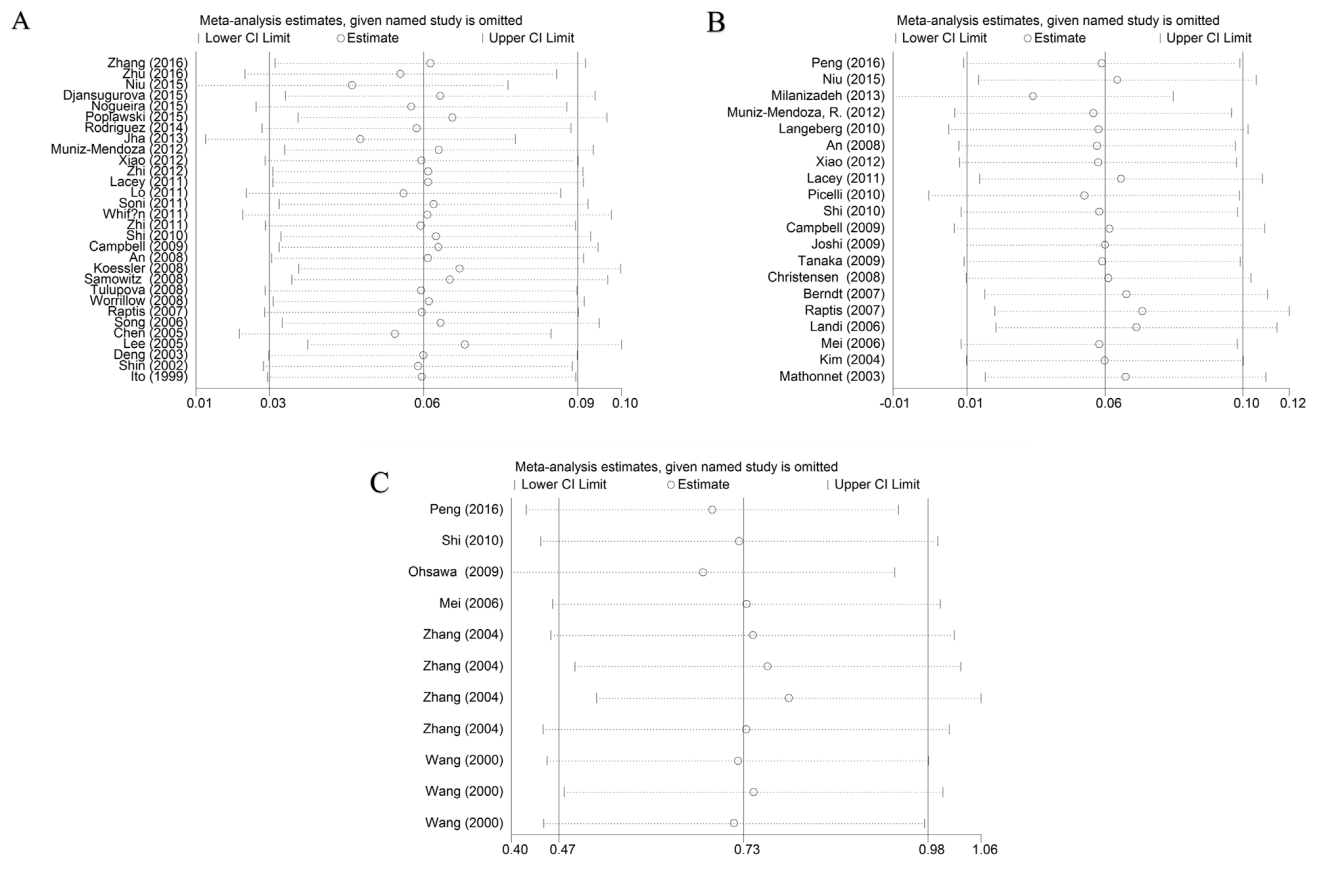
Sensitivity analysis of the associations between hMLH1 polymorphisms and cancer risk according to HWE (**(A)** rs1800734; **(B)** rs1799977; **(C)** rs63750447). HWE: Hardy-Weinberg equilibrium.

As shown in Supplementary Table 1, significant heterogeneities existed after pooled the data of rs1800734 and rs1799977 polymorphisms under different comparison models (*P* ≤ 0.10 or I^2^ ≥ 50%), thus further subgroup analyses base on ethnicity, cancer type, source of control, and quality scores were performed. No obvious heterogeneity was found for rs63750447 polymorphism (*P* > 0.10 or I^2^ < 50%). Subsequent sensitivity analysis proved the stability of our study, since no significant alteration was detected after removing each individual study and rechecking the pooled ORs and 95%CIs for the rs1800734 and rs1799977 polymorphisms (Figure 5A, 5B). The third study performed by Zhang et al seemingly altered the pooled ORs significantly (Figure 5), and the detailed data from Stata 14.0 also showed us it was nearly approached to the upper limit. We guess it was due to the sample size of rs63750447 polymorphism was insufficient, only 11 studies from 6 articles were included. It indicated us the overall results of rs63750447 should be treated more carefully.

### Publication bias

**Figure 6:**
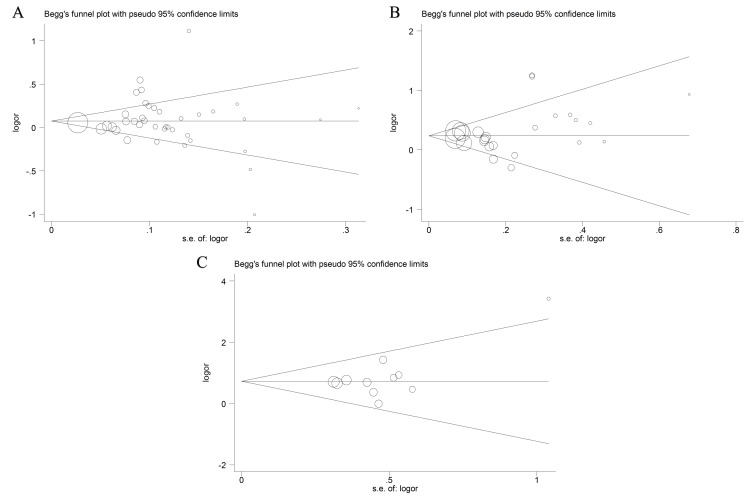
Funnel plots of publication bias (**(A)** rs1800734; **(B)** rs1799977; **(C)** rs63750447).

The possible publication bias in the eligible literature was evaluated by Egger's test and funnel plots. As shown in Figure 6, the Begg's funnel plots appear to be symmetrical. This symmetry was then confirmed by the statistical results of Egger's test (P > 0.05, shown in Table 3). These provided evidence for the absence of publication bias.

**Table 3:** Egger's test for publication bias test of hMLH1 polymorphisms

Egger's test	SE	Coef	Std. Err	t	P>|t|	95%CI
rs1800734	slope	0.06249	0.064308	0.97	0.337	[-0.067807, 0.192794]
	bias	0.15166	0.749679	0.20	0.841	[-1.367335, 1.670654]
rs1799977	slope	0.17888	0.082661	2.16	0.042	[0.007456, 0.350311]
	bias	0.48454	0.597343	0.81	0.426	[-0.754272, 1.723357]
rs63750447	slope	-0.12387	0.497384	-0.25	0.809	[-1.249034, 1.001287]
	bias	2.03105	1.146982	1.77	0.110	[-0.563603, 4.625704]

SE: standard error; 95%CI: 95% confidence interval.

## DISCUSSION

To elucidate the veritable relationship between three hMLH1 polymorphisms (rs1800734; rs1799977; rs63750447) and cancer risk, we performed this meta-analysis based on overall published data up to May 2017. We found all of these polymorphisms can enhance overall cancer risks, especially Asians, under different genetic comparisons (Supplementary Table 1). Further subgroup analyses were carried out according to cancer type, source of control, quality score, and study design, and results worth discussing were obtained.

Interestingly, we found a moderate association existing between rs1800734 and the risk of gastric cancer in three genetic models (OR = 1.14, *P* = 0.017; OR = 1.33, *P* = 0.019; OR = 1.27, *P* = 0.024) and lung cancer in recessive model (OR = 1.27, *P* = 0.024), while no connection was display with colorectal cancer. As far as we know now, microsatellite instability (MSI) often occurs when mismatch errors failed to be corrected or hMLH1 gene was epigenetic silencing. Campbell et al. [41] found rs1800734 polymorphism enhanced MSI-positive colorectal cancer, the association was proved by Mrkonjic et al. [42] due to the effects of rs1800734 on the MLH1 promoter methylation, immunohistochemistry (IHC) deficiency, or both. This indicated us when performing further studies focused on the relationship between rs1800734 and cancer risk, the MSI-statue of cancer patients should be evaluated fundamentally.

Rs1799977 was a nonsynonymous coding polymorphism in hMLH1, which leaded to an amino acid change from isoleucine to valine. The mutational G allele of rs1799977 polymorphism was proved to connect with susceptibility of colorectal cancer and prostate cancer. For rs63750447, the cancer-specific analysis showed an increased risk of colorectal cancer, endometrial cancer and gastric cancer. Recently, rs63750447 was observed over-expressed in patients with EGFR-TKI (epidermal growth factor receptor-tyrosine kinase inhibitor) resistance, which has a possible shorter progression-free survival [43]. Thus, it was speculated that MLH1 might be involved in EGFR signaling or other pathways (such as proliferation and survival) [1].

Compare with previous meta-analyses study on the association between hMLH1 and cancer risk, our study included a larger sample size and performed more detailed stratification analysis. Besides, our study has stricter inclusion criteria and exclude criteria, thus avoided omissive and false drop (refer to the section of Characteristics of eligible studies, paragraph one). Thus, we think our results are more reliable and convinced. Moreover, we found rs1800734 was related to gastric cancer, while rs1799977 may have an influence on colorectal and prostate cancer. It may give us some hints for the further study.

There are still some limitations existing in this meta-analysis. Firstly, insufficiency of original data limited us to proceed more accurate analyses on the potential interaction between these polymorphisms and other risk factors such as age, sex, hereditary background, lifestyle, and MSI status, etc. Secondly, the studies involved in the rs63750447 analysis was insufficient, whose statistical significance was needed to verify by further well-designed study with larger sample sizes. Thirdly, we couldn't exclude the publication bias absolutely according to the negative results of Egger's test and funnel plots. Fourthly, the sample size was still small for any given cancer type, although we have pooled all published literatures. Hence, all the three hMLH1 polymorphisms were associated with cancer risk, but further profoundly investigation was requisite to clarify the strength of these associations.

## MATERIALS AND METHODS

PRISMA statement was used to guide the process of this meta-analysis [44].

### Search strategy

A comprehensive literature search was conducted using the following search terms: (“cancer”, “carcinoma”, “tumor”, “tumour”, or “neoplasm”) and (“polymorphism”, “variation”, “variant”, or “mutation”) and (“hMLH1”). The PubMed, Web of knowledge, VIP, WanFang and Chinese National Knowledge Infrastructure (CNKI) databases were searched up to May, 2017. Additional studies were identified by manual search of the references of the original studies or review articles. This study was approved by the ethics committee of Xi’an Jiaotong University.

To be eligible for this meta-analysis, the included study was required to (1) be case-control or case-cohort studies; (2) focused on the relationship between hMLH1 polymorphisms and risk of any cancer; (3) have at least three articles for each studied hMLH1 polymorphism, and available information concerning the genotype frequency of each included SNP of hMLH1 (i.e., rs1800734; rs1799977; rs63750447); (4) be published in English or Chinese. The exclusion criteria were as follows: (1) studies were not focused on cancer risk or targeted hMLH1 SNPs (rs1800734; 2: rs1799977; 3: rs63750447); (2) studies failed to supply any data on genotype distribution, (3) studies were updated by a following study where a larger number of subjects were included, (4) studies were designed as a case-case or case-only study. If 2 or more studies contained overlapping data, we selected the paper included more samples. Studies containing two or more case-control groups were considered as two or more independent studies.

### Data extraction and quality assessment

For each included study, two investigators independently extracted the raw data and demographic information, including publication year, first author, ethnicity and country or origin, the number of cases and controls, source of controls, genotyping methods, genetic distribution, and P value of Hardy-Weinberg equilibrium (HWE) among the controls. Studies not follow HWE were excluded in subgroup analysis. We applied the Newcastle-Ottawa Scale (NOS) to evaluate the methodological quality of the eligible studies according to Zeng et al [45]. Accumulated score ranges from 0 to 9 points, and a score of 0-5 and 6-9 is considered to suggest a low and high quality respectively, with higher quality representing lower risks of bias. A discussion or consultation with a senior author was conducted to settle controversy until a consensus was reached.

### Statistical analysis

To evaluate the strength of association between hMLH1 polymorphisms (rs1800734; rs1799977; rs63750447) and cancer risk, we calculated the odds ratios (ORs) and 95% confidence intervals (CIs) based on the genotype and allele frequencies in cases and controls of each eligible study. We used the Z test to access the significance of all pooled ORs and it was considered statistically significant if the P value < 0.05. The Chisquare-based Q statistic test and I^2^ statistic were applied to examine the statistical heterogeneity among studies. When no obvious heterogeneity existed across the studies (P>0.10 or I^2^ <50%), we pooled the ORs using fixed-effect model (Mantel– Haenszel); otherwise, the random effects model (DerSimonian and Laird) was chosen. The potential publication bias was evaluated by funnel plot and Egger's test. To access the stability of the results in this meta-analysis, we performed sensitivity analysis by sequentially excluding each study and rechecked whether the pooled ORs were altered significantly.

The following genetic models were evaluated: allele comparison (B vs. A), homozygote comparison (BB vs. AA), heterozygote comparison (AB vs. AA), recessive model (BB vs. AA+ AB), and dominant model (BB+ AB vs. AA). “A” represents the wild allele, while “B” represents the mutation allele. After excluded studies not according to HWE, we conducted the subgroup analysis based on ethnicity (divided into Asian and Caucasian), cancer type, and source of control. All statistical analyses were calculated with the software STATA (Version 14.0; Stata Corp, College Station, TX).

## SUPPLEMENTARY MATERIALS TABLE




