# Supplementary material for: Pooling-analysis on hMLH1 polymorphisms and cancer risk: evidence based on 31,484 cancer cases and 45,494 cancer-free controls
Source: Oncotarget. 2017 Oct 10;8(54):93063–78. doi: 10.18632/oncotarget.21810 (PMC5696244; doi:10.18632/oncotarget.21810)
Supplement: Supplementary file 1 [file oncotarget-08-93063-s001.pdf]

Pooling-analysis on hMLH1 polymorphisms and cancer risk: evidence based on 31,484 cancer cases and 45,494 cancer-free controls

SUPPLEMENTARY TABLE

Supplementary Table 1: Meta-analysis of the associations between hMLH1 polymorphisms and cancer risk

See Supplementary File 1
