# Supplementary material for: Pooling-analysis on hMLH1 polymorphisms and cancer risk: evidence based on 31,484 cancer cases and 45,494 cancer-free controls
Source: Oncotarget. 2017 Oct 10;8(54):93063–78. doi: 10.18632/oncotarget.21810 (PMC5696244; doi:10.18632/oncotarget.21810)
Supplement: Supplementary file 2 [file oncotarget-08-93063-s002.docx]

**Supplementary Table 1: Meta-analysis of the associations between hMLH1 polymorphisms and cancer risk**.

| **Comparisons** | **No. of studies** | **OR** | **95%CI** | **P value** | **Heterogeneity** | | **Effects model** |
| --- | --- | --- | --- | --- | --- | --- | --- |
|  |  |  |  |  | **I^2^** | **P value** |  |
| **-93G>A (rs1800734)** | | | | | | | |
| **A vs. G** | 39 | **1.08** | **1.01-1.16** | **0.023** | 80.70% | <0.001 | R |
| HWE | 30 | 1.06 | 0.99-1.15 | 0.113 | 81.40% | <0.001 | R |
| Asian | 17 | **1.16** | **1.03-1.31** | **0.014** | 84.00% | <0.001 | R |
| Caucasian | 11 | 0.97 | 0.89-1.06 | 0.531 | 67.50% | 0.001 | R |
| CRC | 11 | 1.07 | 0.97-1.17 | 0.174 | 76.40% | <0.001 | R |
| GC | 4 | **1.14** | **1.02-1.28** | **0.017** | 0.00% | 0.585 | F |
| OC | 2 | 0.97 | 0.87-1.08 | 0.570 | 49.10% | 0.161 | F |
| HNSCC | 2 | 1.90 | 0.76-4.74 | 0.167 | 96.30% | <0.001 | R |
| EC | 2 | 0.61 | 0.23-1.65 | 0.335 | 94.40% | <0.001 | R |
| LC | 2 | 1.11 | 0.99-1.24 | 0.078 | 7.70% | 0.298 | F |
| Hospital | 17 | 1.01 | 0.87-1.16 | 0.918 | 82.10% | <0.001 | R |
| population | 13 | **1.10** | **1.00-1.20** | **0.041** | 81.90% | <0.001 | R |
| quality score ≥ 6 | 26 | **1.10** | **1.02-1.18** | **0.013** | 79.40% | <0.001 | R |
| quality score < 6 | 4 | 0.75 | 0.42-1.36 | 0.345 | 87.40% | <0.001 | R |
| digestive cancer | 16 | **1.10** | **1.02-1.19** | **0.017** | 70.60% | <0.001 | R |
| **AA vs. GG** | 39 | **1.23** | **1.06-1.42** | **0.006** | 73.70% | <0.001 | R |
| HWE | 30 | **1.18** | **1.00-1.39** | **0.048** | 76.80% | <0.001 | R |
| Asian | 17 | **1.37** | **1.09-1.72** | **0.008** | 79.20% | <0.001 | R |
| Caucasian | 11 | 0.99 | 0.80-1.23 | 0.929 | 63.40% | 0.002 | R |
| CRC | 11 | 1.12 | 0.90-1.40 | 0.301 | 72.20% | <0.001 | R |
| GC | 4 | **1.33** | **1.06-1.68** | **0.019** | 0.00% | 0.461 | F |
| OC | 2 | 1.04 | 0.78-1.38 | 0.794 | 0.00% | 0.384 | F |
| HNSCC | 2 | 3.22 | 0.75-13.76 | 0.115 | 92.70% | <0.001 | R |
| EC | 2 | 0.14 | 0.00-16.18 | 0.414 | 94.50% | <0.001 | R |
| LC | 2 | 1.27 | 0.96-1.67 | 0.041 | 27.50% | 0.240 | R |
| Hospital | 17 | 1.10 | 0.83-1.48 | 0.503 | 78.30% | <0.001 | R |
| population | 13 | **1.22** | **1.00-1.49** | **0.050** | 76.50% | <0.001 | R |
| quality score ≥ 6 | 26 | **1.23** | **1.05-1.44** | **0.010** | 75.00% | <0.001 | R |
| quality score < 6 | 4 | 0.42 | 0.01-1.76 | 0.233 | 87.20% | <0.001 | R |
| digestive cancer | 16 | **1.21** | **1.02-1.44** | **0.033** | 66.70% | <0.001 | R |
| **GA vs. GG** | 39 | 1.03 | 0.95-1.11 | 0.521 | 66.30% | <0.001 | R |
| HWE | 30 | 1.03 | 0.99-1.07 | 0.181 | 49.40% | 0.001 | R |
| Asian | 17 | 1.05 | 0.92-1.21 | 0.450 | 60.40% | 0.001 | R |
| Caucasian | 11 | 0.98 | 0.93-1.05 | 0.609 | 4.00% | 0.405 | F |
| CRC | 11 | 1.04 | 0.99-1.09 | 0.121 | 33.10% | 0.134 | F |
| GC | 4 | 1.23 | 0.99-1.53 | 0.063 | 0.00% | 0.789 | F |
| OC | 2 | 0.83 | 0.57-1.20 | 0.313 | 70.80% | 0.064 | R |
| HNSCC | 2 | 1.54 | 0.85-2.79 | 0.155 | 80.40% | 0.024 | R |
| EC | 2 | 0.92 | 0.69-1.21 | 0.526 | 0.00% | 0.774 | F |
| LC | 2 | 1.00 | 0.84-1.20 | 0.962 | 0.00% | 0.797 | F |
| Hospital | 17 | 0.93 | 0.80-1.09 | 0.390 | 56.10% | 0.002 | R |
| population | 13 | 1.04 | 0.99-1.09 | 0.070 | 36.30% | 0.092 | F |
| quality score ≥ 6 | 26 | 1.03 | 0.95-1.10 | 0.509 | 53.50% | 0.001 | R |
| quality score < 6 | 4 | 0.81 | 0.57-1.14 | 0.221 | 0.00% | 0.668 | F |
| digestive cancer | 16 | **1.05** | **1.01-1.10** | **0.031** | 29.90% | 0.125 | F |
| **AA+GA vs. GG** | 39 | 1.07 | 0.99-1.16 | 0.110 | 73.50% | <0.001 | R |
| HWE | 30 | 1.06 | 0.97-1.15 | 0.219 | 69.40% | <0.001 | R |
| Asian | 17 | 1.15 | 0.97-1.35 | 0.102 | 76.70% | <0.001 | R |
| Caucasian | 11 | 0.99 | 0.93-1.06 | 0.929 | 24.70% | 0.208 | F |
| CRC | 13 | 1.04 | 0.95-1.15 | 0.366 | 59.30% | 0.006 | R |
| GC | 4 | **1.27** | **1.03-1.56** | **0.024** | 0.00% | 0.620 | F |
| OC | 2 | 0.86 | 0.61-1.20 | 0.364 | 69.20% | 0.072 | R |
| HNSCC | 2 | 1.99 | 0.73-5.38 | 0.177 | 94.00% | <0.001 | R |
| EC | 2 | 0.74 | 0.36-1.49 | 0.396 | 63.30% | 0.099 | R |
| LC | 2 | 1.07 | 0.90-1.26 | 0.432 | 0.00% | 0.540 | F |
| Hospital | 17 | 1.03 | 0.94-1.12 | 0.569 | 70.40% | <0.001 | R |
| population | 13 | **1.05** | **1.01-1.10** | **0.016** | 70.30% | <0.001 | R |
| quality score ≥ 6 | 26 | 1.07 | 0.99-1.17 | 0.103 | 71.00% | <0.001 | R |
| quality score < 6 | 4 | 0.76 | 0.55-1.05 | 0.186 | 40.40% | 0.169 | F |
| digestive cancer | 16 | 1.10 | 1.00-1.20 | 0.049 | 56.90% | 0.003 | R |
| **AA vs. GA+GG** | 39 | **1.22** | **1.09-1.37** | **0.001** | 69.60% | <0.001 | R |
| HWE | 30 | **1.18** | **1.04-1.34** | **0.012** | 70.70% | <0.001 | R |
| Asian | 17 | **1.30** | **1.11-1.53** | **0.001** | 72.80% | <0.001 | R |
| Caucasian | 11 | 1.02 | 0.83-1.24 | 0.873 | 60.60% | 0.005 | R |
| CRC | 13 | 1.14 | 0.94-1.38 | 0.175 | 70.30% | 0.003 | R |
| GC | 4 | 1.15 | 0.98-1.36 | 0.089 | 0.00% | 0.688 | F |
| OC | 2 | 1.10 | 0.83-1.46 | 0.503 | 0.00% | 0.744 | F |
| HNSCC | 2 | 2.56 | 0.85-7.74 | 0.096 | 89.00% | 0.003 | R |
| EC | 2 | 0.15 | 0.00-25.11 | 0.465 | 95.80% | <0.001 | R |
| LC | 2 | **1.27** | **1.03-1.57** | **0.024** | 29.10% | 0.235 | F |
| Hospital | 17 | **1.19** | **1.08-1.31** | **0.000** | 72.40% | <0.001 | R |
| population | 13 | **1.12** | **1.04-1.22** | **0.004** | 69.10% | <0.001 | R |
| quality score ≥ 6 | 26 | **1.18** | **1.11-1.25** | **0.000** | 66.00% | <0.001 | R |
| quality score < 6 | 4 | **0.61** | **0.44-0.86** | **0.004** | 90.00% | <0.001 | R |
| digestive cancer | 16 | **1.17** | **1.02-1.33** | **0.023** | 58.70% | 0.002 | R |
| **655A>G(rs1799977)** | | | | | | | |
| **G vs. A** | 23 | **1.12** | **1.02-1.23** | **0.017** | 69.90% | <0.001 | R |
| HWE | 19 | 1.11 | 0.99-1.24 | 0.057 | 71.90% | <0.001 | R |
| Asian | 9 | **1.64** | **1.38-1.95** | **0.000** | 65.80% | 0.003 | R |
| Caucasian | 10 | 1.03 | 0.98-1.08 | 0.300 | 39.80% | 0.092 | F |
| CRC | 10 | **1.21** | **1.03-1.42** | **0.023** | 80.70% | <0.001 | R |
| PC | 2 | 1.08 | 0.96-1.22 | 0.202 | 0.00% | 0.536 | F |
| LC | 2 | 1.20 | 0.48-2.96 | 0.698 | 82.40% | 0.017 | R |
| Hospital | 8 | 1.32 | 0.89-1.94 | 0.164 | 86.40% | <0.001 | R |
| population | 11 | 1.05 | 1.00-1.10 | 0.078 | 4.50% | 0.400 | F |
| case-control | 16 | **1.17** | **1.01-1.36** | **0.038** | **75.80%** | **<0.001** | **R** |
| case-cohort | 3 | 1.05 | 0.97-1.14 | 0.228 | 1.40% | 0.363 | F |
| **GG vs. AA** | 22 | **1.15** | **1.04-1.27** | **0.006** | 49.80% | 0.004 | F |
| HWE | 18 | **1.13** | **1.01-1.26** | **0.027** | 47.40% | 0.014 | F |
| Asian | 8 | **3.44** | **2.12-5.59** | **0.000** | **0.00%** | 0.980 | F |
| Caucasian | 10 | 1.06 | 0.95-1.19 | 0.308 | 6.60% | 0.381 | F |
| CRC | 9 | 1.26 | 0.94-1.70 | 0.119 | 68.50% | 0.001 | R |
| PC | 2 | 1.09 | 0.83-1.45 | 0.529 | 0.00% | 0.421 | F |
| LC | 2 | 0.75 | 0.42-1.34 | 0.529 | 0.00% | 0.374 | F |
| Hospital | 8 | 1.88 | 0.84-4.24 | 0.127 | 72.60% | 0.001 | R |
| population | 10 | 1.08 | 0.96-1.22 | 0.276 | 0.00% | 0.891 | F |
| case-control | 15 | 1.22 | 0.94-1.59 | 0.140 | 52.40% | 0.009 | R |
| case-cohort | 3 | 1.05 | 0.87-1.27 | 0.622 | 13.90% | 0.313 | F |
| **AG vs. AA** | 23 | 1.04 | 0.98-1.10 | 0.182 | 39.70% | 0.027 | F |
| HWE | 19 | 1.03 | 0.97-1.10 | 0.359 | 22.70% | 0.180 | F |
| Asian | 9 | 1.16 | 0.93-1.45 | 0.183 | 19.00% | 0.274 | F |
| Caucasian | 10 | 1.02 | 0.95-1.09 | 0.578 | 25.70% | 0.207 | F |
| CRC | 10 | 1.04 | 0.96-1.12 | 0.320 | 0.00% | 0.555 | F |
| PC | 2 | 1.13 | 0.96-1.33 | 0.133 | 0.00% | 0.938 | F |
| LC | 2 | 1.10 | 0.44-2.76 | 0.806 | 80.60% | 0.846 | R |
| Hospital | 8 | 0.91 | 0.77-1.08 | 0.289 | 28.30% | 0.202 | F |
| population | 11 | 1.05 | 0.98-1.13 | 0.160 | 11.40% | 0.336 | F |
| case-control | 16 | 1.00 | 0.93-1.08 | 0.997 | 28.70% | 0.135 | F |
| case-cohort | 3 | 1.10 | 0.98-1.23 | 0.099 | 0.00% | 0.841 | F |
| **GG+AG vs. AA** | 24 | **1.28** | **1.16-1.41** | **0.000** | 57.60% | <0.001 | R |
| HWE | 19 | **1.29** | **1.17-1.49** | **0.000** | 65.90% | <0.001 | R |
| Asian | 9 | **1.52** | **1.04-2.24** | **0.033** | 64.50% | 0.004 | R |
| Caucasian | 9 | **1.24** | **1.16-1.32** | **0.000** | 30.40% | 0.175 | F |
| CRC | 10 | **1.36** | **1.18-1.57** | **0.000** | 56.10% | 0.015 | R |
| PC | 2 | **1.36** | **1.16-1.59** | **0.000** | 0.00% | 0.631 | F |
| LC | 2 | 1.16 | 0.56-2.37 | 0.694 | 70.70% | 0.065 | R |
| Hospital | 8 | 1.32 | 0.92-1.89 | 0.136 | 74.90% | <0.001 | R |
| population | 12 | **1.27** | **1.19-1.35** | **0.000** | 0.00% | 0.624 | F |
| case-control | 16 | **1.26** | **1.09-1.45** | **0.002** | 55.90% | 0.003 | R |
| case-cohort | 3 | **1.34** | **1.20-1.50** | **0.000** | 0.00% | 0.685 | F |
| **GG vs. AA+AG** | 22 | 1.21 | 1.00-1.45 | 0.051 | 60.90% | <0.001 | R |
| HWE | 18 | 1.17 | 0.94-1.46 | 0.166 | 63.10% | <0.001 | R |
| Asian | 8 | **3.34** | **2.33-4.78** | **0.000** | 0.00% | 0.984 | F |
| Caucasian | 10 | 1.05 | 0.94-1.17 | 0.369 | 0.00% | 0.517 | F |
| CRC | 9 | 1.29 | 0.92-1.82 | 0.142 | 79.70% | <0.001 | R |
| PC | 2 | 1.03 | 0.79-1.35 | 0.829 | 0.00% | 0.402 | F |
| LC | 2 | 0.87 | 0.50-1.52 | 0.626 | 0.00% | 0.434 | F |
| Hospital | 8 | 1.91 | 0.88-4.15 | 0.105 | 75.70% | <0.001 | R |
| population | 10 | 1.06 | 0.95-1.19 | 0.277 | 0.00% | 0.866 | F |
| case-control | 15 | 1.29 | 0.96-1.74 | 0.091 | 65.80% | 0.000 | R |
| case-cohort | 3 | 1.00 | 0.83-1.20 | 0.991 | 1.20% | 0.363 | F |
| **1151T>A(rs63750447)** | | | | | | | |
| **A vs. T** | 11 | **2.19** | **1.72-2.78** | **0.000** | 24.60% | 0.209 | F |
| CRC | 5 | **2.84** | **1.38-5.81** | **0.004** | 59.70% | 0.042 | R |
| EC | 2 | **1.80** | **1.05-3.09** | **0.033** | 0.00% | 0.776 | F |
| GC | 2 | **2.07** | **1.24-3.47** | **0.006** | 0.00% | 0.729 | F |
| quality score ≥ 6 | 8 | **2.21** | **1.70-2.87** | **0.000** | 46.00% | 0.073 | F |
| quality score < 6 | 3 | **2.08** | **1.14-3.76** | **0.016** | 0.00% | 0.836 | F |
| **TA vs. TT** | 11 | **2.21** | **1.73-2.84** | **0.000** | 18.00% | 0.273 | F |
| CRC | 5 | **2.81** | **1.42-5.57** | **0.003** | 54.30% | 0.067 | R |
| EC | 2 | **1.85** | **1.06-3.21** | **0.029** | 0.00% | 0.766 | F |
| GC | 2 | **2.15** | **1.27-3.64** | **0.005** | 0.00% | 0.719 | F |
| quality score ≥ 6 | 8 | **2.23** | **1.69-2.92** | **0.000** | 40.90% | 0.106 | F |
| quality score < 6 | 3 | **2.15** | **1.17-3.95** | **0.014** | 0.00% | 0.823 | F |
| **AA+TA vs. TT** | 11 | **2.23** | **1.75-2.86** | **0.000** | 21.90% | 0.235 | F |
| CRC | 5 | **2.87** | **1.42-5.82** | **0.003** | 57.10% | 0.054 | R |
| EC | 2 | **1.85** | **1.06-3.21** | **0.029** | 0.00% | 0.766 | F |
| GC | 2 | **2.15** | **1.27-3.64** | **0.005** | 0.00% | 0.719 | F |
| quality score ≥ 6 | 8 | **2.25** | **1.72-2.95** | **0.000** | 43.80% | 0.086 | F |
| quality score < 6 | 3 | **2.15** | **1.17-3.95** | **0.014** | 0.00% | 0.823 | F |

HWE: Hardy–Weinberg equilibrium; CRC: colorectal cancer; GC: gastric cancer; OC: ovarian carcinoma; HNSCC: head and neck squamous cell carcinoma; EC: endometrial cancer; LC: lung cancer; PC: prostate cancer
